# Dexmedetomidine Acts via the JAK2/STAT3 Pathway to Attenuate Isoflurane-Induced Neurocognitive Deficits in Senile Mice

**DOI:** 10.1371/journal.pone.0164763

**Published:** 2016-10-21

**Authors:** Yanna Si, Yuan Zhang, Liu Han, Lihai Chen, Yajie Xu, Fan Sun, Muhuo Ji, Jianjun Yang, Hongguang Bao

**Affiliations:** 1 Department of Anesthesiology, Nanjing First Hospital, Nanjing Medical University, Nanjing, Jiangsu, China; 2 Department of Anesthesiology, Zhongda Hospital, School of Medicine, Southeast University, Nanjing, Jiangsu, China; Jiangsu Province Key Laboratory of Anesthesiology, College of Anesthesiology, Xuzhou Medical College, Xuzhou, Jiangsu, China; University of Pennsylvania, UNITED STATES

## Abstract

**Background:**

Previous studies showed that isoflurane-induced cognitive deficits could be alleviated by dexmedetomidine in young animal subjects. In the current study, we examine whether dexmedetomidine could also alleviate isoflurane-induced cognitive deficits in senile animals.

**Methods:**

Senile male C57BL/6 mice (20 months) received dexmedetomidine (50 μg/kg, i.p.) or vehicle 30 minutes prior to isoflurane exposure (1.3% for 4 h). Cognitive function was assessed 19 days later using a 5-day testing regimen with Morris water maze. Some subjects also received pretreatment with α_2_ adrenoreceptor antagonist atipamezole (250 μg/kg, i.p.), JAK2 inhibitor AG490 (15 mg/kg i.p.) or STAT3 inhibitor WP1066 (40 mg/kg i.p.) 30 minutes prior to dexmedetomidine.

**Results:**

Isoflurane exposure increased and reduced the time spent in the quadrant containing the target platform in training sessions. The number of crossings over the original target quadrant was also decreased. Dexmedotomidine attenuated such effects. Effects of dexmedotomidine were reduced by pretreatment with atipamezole, AG490 and WP1066. Increased phosphorylation of JAK2 and STAT3 in the hippocampus induced by isoflurane was augmented by dexmedetomidine. Effects of dexmedetomidine on JAK2/STAT3 phosphorylation were attenuated by atipamezole, AG490 and WP1066. Isoflurane promoted neuronal apoptosis and increased the expression of cleaved caspase-3 and BAD, and reduced Bcl-2 expression. Attenuation of such effects by dexmedotomidine was partially blocked by atipamezole, AG490 and WP1066.

**Conclusion:**

Dexmedetomidine could protect against isoflurane-induced spatial learning and memory impairment in senile mice by stimulating the JAK2/STAT3 signaling pathway. Such findings encourage the use of dexmedetomidine in geriatric patients receiving isoflurane anesthesia.

## Introduction

Post-operative cognitive dysfunction (POCD) is common after major surgery in geriatric patients: it occurs in nearly 40% of elderly surgery patients prior to discharge, and persists beyond three months in about 10% of the patients [1, 2]. POCD is associated with impaired function, premature departure from the workforce, and increased mortality [3].

Several lines of evidence indicate that general anesthesia can induce cognitive deficits in elderly patients undergoing surgeries [4–6]. Inhalation anesthetics (e.g., isoflurane and sevoflurane), widely used in clinical practices, have been proven to exert the potentially deleterious effects on elderly patients. Previous evidence suggest that isoflurane exposure would be a great risk factor for these patients to suffer from POCD [3–5]. Dexmedetomidine, a highly selective α_2_ adrenoreceptor agonist with sedative and analgesic properties, protects neuronal tissue against ischemic cerebral injury [7,8]. It reduces ischemia-induced neuronal apoptosis by up-regulating anti-apoptotic proteins (e.g., Bcl-2 and Mdm-2) and down-regulating pro-apoptotic proteins (e.g., Bax and p53) [9]. In neonatal rats, dexmedetomidine attenuates isoflurane-induced neurocognitive impairment and neuronal apoptosis by activating the α_2_-adrenergic receptor [10], possibly via activation of the PI3K/Akt pathway [8].

This study was designed to examine whether dexmedetomidine could also protective against isoflurane-induced cognitive impairment in senile mice. To probe the mechanisms of action, we used selective ligands for the α_2_-adrenergic receptor, Janus-activated kinase 2 (JAK2) and signal transducer and activator of transcription 3 (STAT3). The JAK2/STAT3 pathway participates in many physiological processes, including inflammation, proliferation, differentiation, and development [11], and it may help drive the survival response of neurons following exposure to cytokines [11,12]. Inactivating STAT3 enhances neuronal death of cytokine-dependent sensory neurons of the nodose ganglion *in vivo* [13]. In an animal model of Alzheimer׳s disease, activating the JAK2/STAT3 pathway improved spatial learning and memory [14].

## Materials and Methods

### Animals

The study protocol was approved by the Animal Ethics Committee of Nanjing Medical University, and all experiments were conducted in strict accordance with the US National Institutes of Health Guide for the Use of Laboratory Animals. All possible efforts were made to minimize animal suffering. Male C57BL/6 mice (20 months of age and weighing 45–65 g) were used in all experiments. Mice were housed in a temperature- and humidity-controlled facility with a 12-hour light-dark cycle.

### Treatments

Mice were pretreated with dexmedetomidine (50 μg/kg, i.p.) or vehicle at 30 min prior to 4-hour exposure to 1.3% isoflurane (in humidified 30% oxygen carrier gas) in a chamber partially submerged in a 37°C water bath [6,8]. Isoflurane was delivered using a vaporizer (Aerrane, Baxter Healthcare, Deerfield, IL, USA). Concentrations of oxygen, carbon dioxide and isoflurane in the chamber were monitored using a monitor from Detex-Ohmeda (Louisville, KY, USA). A group of mice not exposed to isoflurane was included as an additional control. Core body temperature was measured with a rectal probe and maintained at 37 ± 0.5°C using a heating blanket.

In some experiments, animals were pretreated with the α_2_ adrenoreceptor antagonist atipamezole (250 μg/kg, i.p.), the JAK2 inhibitor AG490 (15 mg/kg i.p; Sigma-Aldrich, St Louis, MO, USA) or the STAT3 inhibitor WP1066 (40 mg/kg i.p.; Sigma-Aldrich) at 30 min before dexmedetomidine injection.

Mice were anesthetized with sodium pentobarbital (50 mg/kg, i.p., n = 16) at the end of the 4-hour isoflurane exposure and blood samples were obtained from the femoral artery of animals for measurement of arterial blood gases and blood glucose. Animals were then allowed to recover from anesthesia. Cognition was assessed using a Morris water maze test at 2 w after isoflurane exposure.

### Morris water maze

Cognitive function was assessed using a standard 5-day Morris water maze test as described [15]. The test was performed by a researcher blinded to treatment conditions, starting at 2 weeks after isoflurane exposure. The water maze consisted of a round, painted pool with a diameter of 100 cm and height of 40 cm, which was filled with opaque water to a depth of 28 cm and maintained at 23±1°C. Animals were trained to escape by swimming onto a hidden platform at 0.5 cm beneath the water surface. The room was decorated with visual spatial cues. Sessions were recorded using a video tracking system. Data were analyzed using a motion detection software (Actimetrics Software, Evanston, IL, USA).

The first four days were spatial acquisition training and testing sessions. All mice participated in four sessions per day at an inter-session interval of 20 min with the platform in the third quadrant of the swimming pool. At the beginning of each session, mice were placed into the water in a fixed position facing the pool wall. The maximum length of the sessions was 60 sec. Animals that failed to locate the platform within 60 sec were manually guided to the platform. Mice were allowed to stay on the platform for 20 sec before being removed. After each session, mice were dried before being transferred to the holding cage. Swimming speed and the amount of time spent locating the platform (escape latency) were calculated from the videos using the motion detection software. The escape latency from all four sessions was averaged to calculate the escape latency for each day. On the fifth day, the platform was removed to allow for probe testing. During the 60-sec session, the number of crossings over the platform that had served as the target on days 1–4 was recorded, as was the total time spent in the quadrant where the original target platform was located.

At the end of testing, some mice (n = 8) were euthanized by perfusion with 200-mL normal saline, followed by fixation with 250-mL 4% formaldehyde. The hippocampus was harvested and post-fixed overnight in 4% formaldehyde at 4°C for TUNEL analysis.

Another group of mice (n = 8) were sacrificed at the end of testing and perfused with 100-mL normal saline for analysis of protein expression in the hippocampus. The hippocampus, including CA1 and dentate gyrus, was dissected on ice and stored at −80°C for apoptosis assay and measurement of caspase-3, BDNF, CREB and JAK2/STAT3 expression.

### Apoptosis assay

Paraffin-embedded sections (4 μm) of the hippocampal CA1 region were deparaffinized, rehydrated, and permeabilized with proteinase K. Slides were incubated in equilibration buffer for 20 min at 95°C, then stained using a TACS 2 TdT-DAB *in situ* apoptosis detection kit (Trevigen, Gaithersburg, MD, USA). Five fields were randomly selected for each slide, and at least 200 cells were counted at 400x magnification by a researcher blinded to treatment conditions. The percentage of apoptosis was estimated using the formula: percent apoptosis = (number of TUNEL-positive cells/total number of cells) × 100%.

### Western blotting

Hippocampus was homogenized and centrifuged at 8000 *g* for 20 min. The supernatant was separated by 12% SDS-PAGE, then transferred to a nitrocellulose membrane (Hybond-ECL, Amersham Biosciences, Little Chalfont, UK). Membranes were blocked, then incubated overnight at 4°C with antibodies (1:1000; Santa Cruz Biotechnology, Santa Cruz, CA, USA) against JAK2, phosphorylated JAK2 (p-JAK2), STAT3, p-STAT3, brain-derived neurotrophic factor (BDNF), cAMP response element-binding protein (CREB), p-CREB, BAD, or caspase-3; or with antibodies (1:2000; Santa Cruz Biotechnology) against Bcl-2 and β-actin. Next, blots were incubated with secondary antibodies conjugated to horseradish peroxidase. Protein bands were detected using enhanced chemiluminescence (Amersham Biosciences) and quantitated using Quantity One software (Bio-Rad Laboratories, UK). Band intensities were normalized to that of β-actin.

### Statistical analysis

All data are expressed as mean ± standard deviation (SD). Statistical analysis was carried out using SPSS 15.0 (IBM, Chicago, IL, USA). Comparisons among multiple groups were conducted using one-way ANOVA, followed by Tukey’s multiple testing. Data from the training sessions were analyzed using two-way ANOVA of repeated measures followed by Bonferroni multiple-comparison for main effects. P< 0.05 was defined as the significance level.

## Results

### Dexmedetomidine attenuated isoflurane-induced cognitive performance

**Fig 1:**
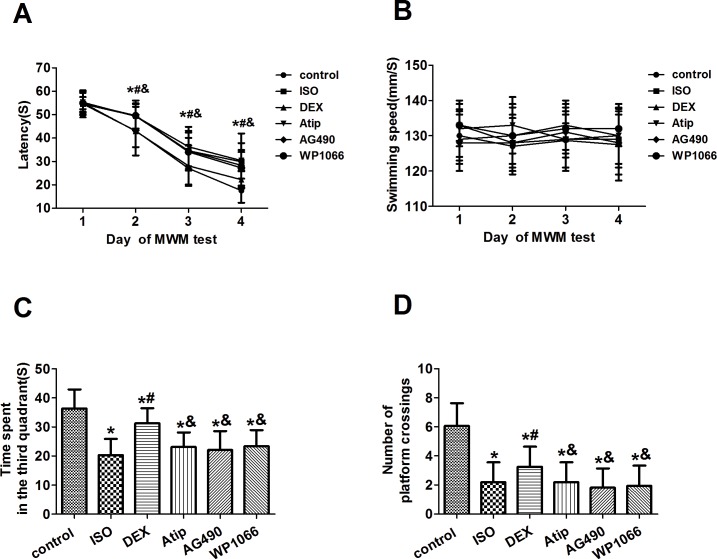
Effects of dexmedetomidine pretreatment on isoflurane-induced behavioral deficits. Latency for mice to reach the target platform (A) and swimming speed (B) were measured during spatial acquisition training and testing on days 1–4. On day 5, the number of crossings over the original target platform (C) and the time spent in that quadrant (D) were measured. Data are represented as mean ± SD (n = 16). *P < 0.05 vs. control group; ^#^*P* < 0.05 vs. isoflurane (ISO) group; ^&^*P* <0.05 vs. dexmedetomidine (DEX) group.

All rats survived the experiments. All treatment groups showed similar escape latencies on day 1 of the Morris water maze testing (Fig 1A, S1 Table). The latency rapidly declined in all treatment groups over days 2–4. Animals not exposed to dexmedetomidine or isoflurane showed latency of 43 ± 7 sec on day 2, 27 ± 7 sec on day 3 and 18 ± 5 sec on day 4. Animals exposed only to isoflurane showed significantly longer latency of 49 ± 5 sec on day 2, 35 ± 8 sec on day 3 and 29 ± 4 sec on day 4 (*P*<0.01). Dexmedetomidine pretreatment attenuated the effects of isoflurane on days 2–4 (*P*<0.05); such effects were attenuated by atipamezole, AG490 or WP1066 (Fig 1A). None of the treatments affected swimming speed (Fig 1B, S2 Table).

On the fifth day of the behavioral testing (Fig 1C and 1D, S3 Table), mice exposed to isoflurane spent less time in the quadrant that had contained the target platform during training (20 ± 6 vs. 36 ± 7 sec in mice not exposed; *P*<0.01). Animals exposed to isoflurane also had lower number of crossing over the original target platform (2.2 ± 1.4 vs. 6.1 ± 1.6; *P*<0.01). Effects of isoflurane exposure were attenuated by dexmedetomidine: time spent in the quadrant that had contained the target platform during training was 31 ± 5 sec, number of crossing was 3.3 ± 1.4 (*P*<0.05 vs. isoflurane alone). Pretreatment with atipamezole, AG490 or WP1066 led to significantly shorter time spent in the quadrant that had contained the target platform during training (23 ± 5, 22 ± 7 or 23 ± 6 sec, respectively; *P*<0.05 vs. control plus dexmedetomidine for all), as well as to significantly fewer platform crossings (2.2 ± 1.4, 1.8 ± 1.3 or 1.9 ± 1.4, respectively; all *P*<0.05).

### Dexmedetomidine attenuated isoflurane-induced apoptosis in the hippocampus

**Fig 2:**
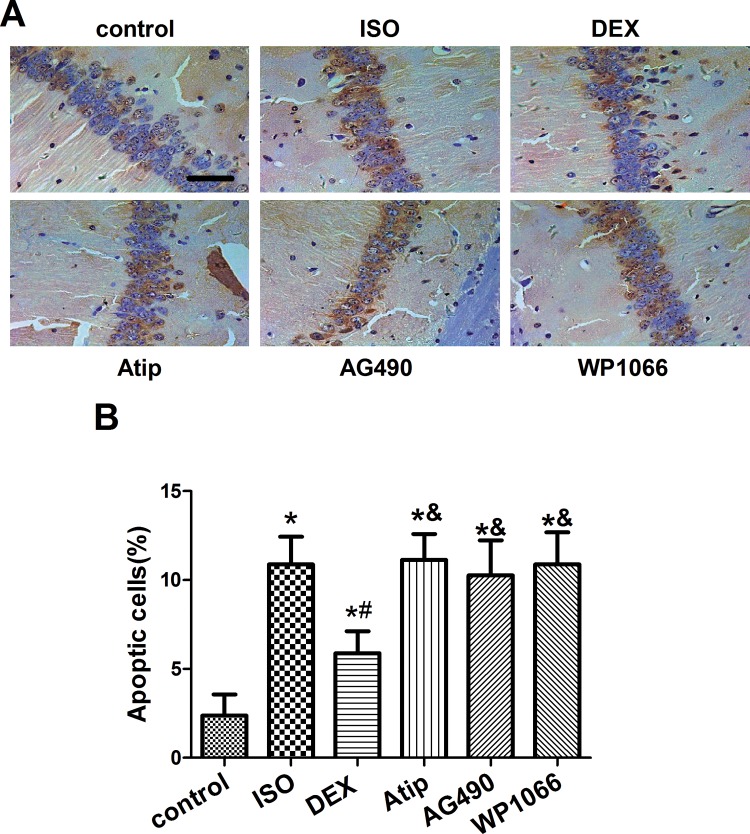
Effects of dexmedetomidine on isoflurane-induced apoptosis in hippocampus. Representative photomicrographs of the hippocampal CA1 region from senile mice at 19 d after treatment with nothing (control) or with isoflurane (ISO) following pretreatment with nothing or with dexmedetomidine (DEX). Some animals were pretreated with atipamezole (Atip), AG490 or WP1066 prior to DEX. Apoptosis was assessed using terminal deoxynucleotidyl transferase dUTP nick end (TUNEL) staining (A), and TUNEL-positive cells were counted (B). Scale bar, 50 μm. Data are represented as mean ± SD (n = 8). *P< 0.05 vs. control group; ^#^*P*< 0.05 vs. ISO group; ^&^*P*< 0.05 vs. DEX group.

Isoflurane exposure increased the number of TUNEL-positive cells in the hippocampal CA1 region (Fig 2A and 2B, S4 Table), and this increase was partially reversed by dexmedetomidine pretreatment (*P*<0.05). These effects of dexmedetomidine were suppressed by atipamezole, AG490 or WP1066 (*P*<0.05 vs. dexmedetomidine).

### Dexmedetomidine attenuated isoflurane-induced changes in caspase-3, BAD and Bcl-2

**Fig 3:**
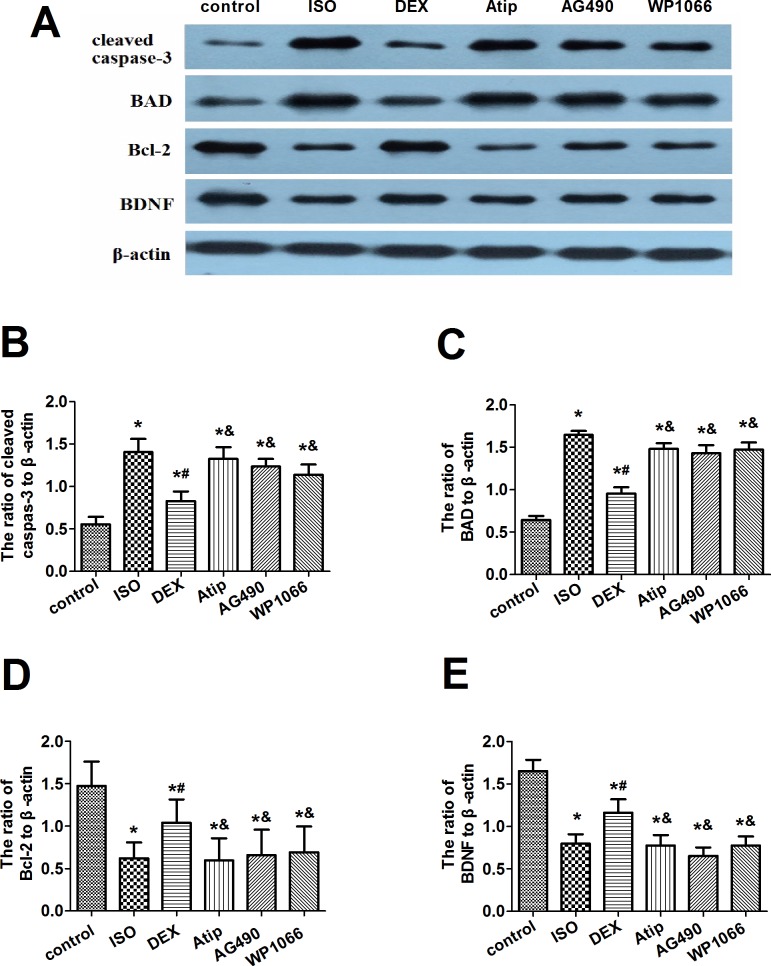
Effects of dexmedetomidine on isoflurane-induced changes in expression of apoptosis-related proteins. Representative Western blots (A) showing levels of cleaved caspase-3, BAD, Bcl-2 and BDNF in hippocampus at 19d after animals were treated as described in Fig 2. Levels of caspase-3 (B), BAD (C), Bcl-2 (D) and BDNF (E) were quantitated relative to that of β-actin. Data are represented as mean ± SD (n = 8). *P < 0.05 vs. control group; ^#^*P*< 0.05 vs. ISO group; ^&^*P*< 0.05 vs. DEX group.

Western blotting showed that isoflurane exposure significantly increased expression of cleaved caspase-3 and BAD but decreased expression of Bcl-2 (Fig 3A–3D, S5 Table). These changes were partially reversed by dexmedetomidine, and this reversal was partially blocked by pretreatment with atipamezole, AG490 or WP1066.

### Dexmedetomidine increased levels of p-JAK2 and p-STAT3

**Fig 4:**
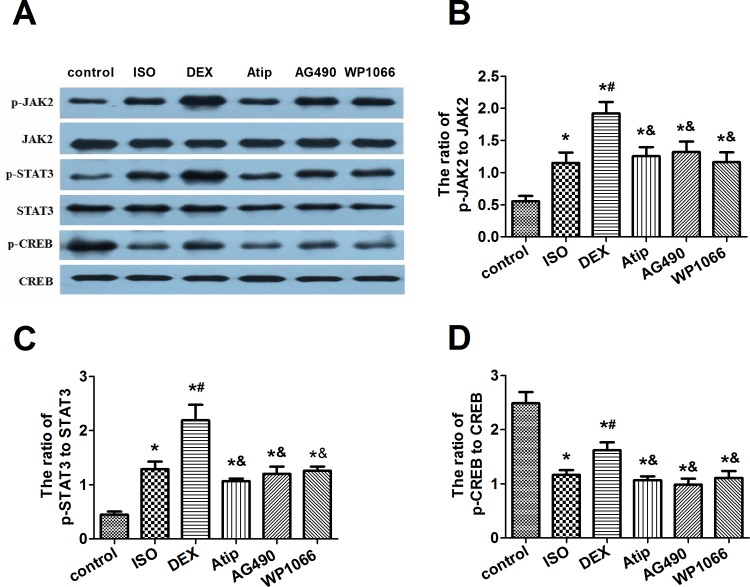
Effects of dexmedetomidine on p-CREB levels and JAK2/STAT3 pathway activation in hippocampus. Representative Western blots (A) showing levels of JAK2, p-JAK2, STAT3, p-STAT3, CREB, and p-CREB in hippocampus at 19d after animals were treated as described in Fig 2. Densitometry was used to determine ratios of the levels of p-JAK2 to JAK2 (B), p-STAT3 to STAT3 (C), and p-CREB to CREB (D). Data are represented as mean ± SD (n = 8). *P < 0.05 vs. control group; ^#^*P*< 0.05 vs. ISO group; ^&^*P*< 0.05 vs. DEX group.

Isoflurane increased phosphorylation of JAK2 and STAT3 in hippocampus (Fig 4A–4C, S6 Table), and dexmedetomidine pretreatment further increased this phosphorylation. This enhancement by dexmedetomidine was attenuated by atipamezole, AG490 or WP1066. None of these treatments significantly affected total levels of JAK2 and STAT3.

### Dexmedetomidine increased levels of p-CREB and expression of BDNF

Since CREB-mediated transcription may increase BDNF expression, we examined levels of both the active form of CREB (p-CREB) and BDNF in the hippocampus. Isoflurane exposure decreased levels of both proteins, and dexmedetomidine pretreatment partially reversed these decreases (Fig 3A and 3E and Fig 4A and 4D, S5 and S6 Tables). The extent of this reversal was smaller after pretreatment with atipamezole, AG490 or WP1066. None of the treatments significantly affected total levels of CREB in hippocampus.

### Isoflurane and dexmedetomidine exerted minimal effects on physiological parameters of senile mice

None of the treatments led to arterial blood gas values outside normal physiological ranges, and these values were similar across all groups (Table 1 and S7 Table). Isoflurane did significantly increase blood glucose levels (*P*<0.05 vs. control), and this increase was significantly smaller after dexmedetomidine pretreatment (*P*<0.05 vs. isoflurane alone). The effects of dexmedetomidine were attenuated by atipamezole (*P*<0.05) and partially reversed by AG490 or WP1066 (*P*<0.05).

**Table 1:** Analysis of arterial blood gases and blood glucose in senile mice immediately after isoflurane exposure.

Group	pH	pO_2_ (mmHg)	pCO_2_ (mmHg)	Blood glucose (mmol/L)
control	7.42±0.05	100.1±6.2	41.5±2.5	5.17±0.67
ISO	7.40±0.07	105.3±8.2	39.7±3.7	8.99±0.83 *
DEX	7.37±0.10	102.7±7.4	42.1±2.9	6.12±0.72 *^#^
Atip	7.36±0.09	104.2±6.7	41.6±2.5	9.04±0.91 *^&^
AG490	7.38±0.11	103.9±7.5	40.8±3.1	8.13±0.61 *^#^^&^
WP1066	7.39±0.08	105.0±6.9	41.9±3.4	7.82±0.77 *^#^^&^

Groups were treated as described in Methods. Data are represented as mean ± SD (n = 16).

* *P* < 0.05 vs. control group

^#^ *P* < 0.05 vs. ISO group

^&^ *P* < 0.05 vs. DEX group.

## Discussion

Dexmedetomidine is used as an adjunct treatment in many clinical applications because of its hypnotic, sedative and analgesic effects. It has also been shown to attenuate apoptosis in the heart, kidneys, brain, intestine and testis in several *in vivo* and *in vitro* models [16]. Studies in neonatal animals suggest that it can reduce apoptosis in hippocampus and impairment of spatial memory induced by isoflurane anesthesia [17]. Here we extend those findings by showing that dexmedetomidine can also reduce isoflurane-induced cognitive dysfunction in senile animals, based on Morris water maze testing. We further show that these effects of dexmedetomidine correlate with its ability to attenuate isoflurane-induced increases in expression of cleaved caspase-3 and BAD, as well as isoflurane-induced decreases in Bcl-2, BDNF and p-CREB. Dexmedetomidine partially reversed isoflurane-induced increases in apoptosis in the hippocampus, based on TUNEL assays. Finally, we show that the effects of dexmedetomidine were reversed by α_2_ adrenoreceptor antagonist atipamezole, the specific JAK2 inhibitor AG490 and the STAT3 inhibitor WP1066. This is the first evidence that the neuroprotective effects of dexmedetomidine in senile mice involve the JAK2/STAT3 pathway.

Hippocampus is a critical brain area for learning, memory, as well as other fundamental cognitive abilities [8,10]. We therefore chose to focus on the hippocampus in the current study. Isoflurane exposure could induce spatial learning and memory impairment [18,19]. In the current study, we assessed cognition using a hippocampus-dependent behavioral test: the Morris water maze test [20,21]. Training sessions were used to evaluate the animals’ ability to learn spatial locations, while probe trials were performed to investigate memory retention. Exposure to a clinically relevant concentration of isoflurane for 4 h resulted in deficits in spatial learning and memory, which manifested as longer escape latency to mount the platform, less time spent in the quadrant where the target platform had been during training and fewer crossings over that original target platform. Extrapolation of the results to other inhalation anesthetics must be careful. However, cognitive deficits induced by varying classes of general anesthetics, such as isoflurane, desflurane and propofol, are similar in many aspects [22–24]. Our finding that dexmedetomidine pretreatment could attenuate isoflurane-induced deficits in spatial learning and memory is consistent with previous work showing that dexmedetomidine attenuates cerebral ischemia/reperfusion injury [9] and partially reverses neurocognitive damage in neonatal rats exposed to a much lower dose of isoflurane [10]. The α_2_ adrenoreceptor antagonist atipamezole reversed the neuroprotective effects of dexmedetomidine in the current study. Additionally, dexmedetomidine has been showed to ameliorate cognitive impairment induced by other anesthesia agents. A recent research reported that dexmedetomidine alleviates delirium after desflurane anesthesia in children [25]. With regards to molecular mechanisms, a previous study showed that dexmedetomidine could ameliorate neurocognitive impairment induced by repeated propofol exposure via the PI3K/Akt/GSK‑3β signaling pathway in neonatal rats [26].

Studies suggest that changes in cerebral structure and neurocognitive defects induced by isoflurane anesthesia result from changes in expression of activated caspase-3 and induction of neural cell apoptosis [27, 28]. Prolonged exposure to isoflurane may also lead to neurotoxicity by inducing excessive Ca^2+^ release from the endoplasmic reticulum [29–31]. Intracellular Ca^2+^ overload activates intrinsic mitochondrion-dependent apoptosis, which appears to be an early event in neuronal impairment [32–35]. In addition, isoflurane inhalation induces overproduction of oxygen free radicals, which damage mitochondria, ultimately triggering cell apoptosis and necrosis [36]. Consistent with these previous studies, we found that exposing senile mice to isoflurane for 4 h increased the number of TUNEL-positive nuclei and caspase-3 expression in the hippocampus. We further found that a single dose of 50 μg/kg dexmedetomidine attenuated neuronal injury induced by inhalation anesthesia, and that this effect was partially reversed by an α_2_ adrenoreceptor antagonist. We were able to exclude significant effects of isoflurane or dexmedetomidine treatment on physiological parameters in the mice, strongly suggesting that dexmedetomidine exerts neuroprotective effects by preventing central neuronal apoptosis.

These findings extend previous work showing that dexmedetomidine pretreatment protects against isoflurane-induced neurocognitive impairment in a dose-dependent way in neonatal rats [8,10]. It also extends *in vitro* studies showing that dexmedetomidine reduces glutamate-induced apoptotic and necrotic cell death in cortical neuron cultures, and that it protects organotypic hippocampal slice cultures against injury caused by hypoxia, abnormal glucose level, ischemia injury or hypercarbia [7,37]. However, the mechanisms of POCD are highly complex, and may include the direct effects of inhalation anesthetics, the consequences of surgical trauma, as well as inflammatory responses. Specific to inflammatory contribution, previous evidence have shown that changes of the level of pro-inflammatory factor and anti-inflammatory factor in hippocampus are relevant to isoflurane-induced cognitive dysfunction [38]. There are also some evidence suggesting that inflammation suppression contributes to alleviation of POCD in elderly patients by dexmedetomidine [39].

Several lines of evidence suggest that the balance of anti- and pro-apoptotic proteins, generally represented by the ratio of Bcl-2/BAD or Bcl-2/BAX, plays a critical role in regulating neuronal apoptosis [40,41]. Proteins of the Bcl-2 family, such as Bcl-2, BAX and BAD, regulate the release of apoptogenic factors from mitochondria as well as regulate mitochondrial membrane integrity [42,43]. The anti-apoptotic protein Bcl-2 plays a pivotal role in regulating neuronal apoptosis under many adverse conditions such as neurodegenerative diseases, ischemia, oxidative stress and trauma. Pro-apoptotic BAD is essential for inducing apoptosis and necrosis in many neuronal populations. Animals deficient in Bcl-2 exhibit massive neural cell apoptosis mediated by the mitochondrial apoptosis pathway. This massive apoptosis can be largely avoided by co-deleting Bax, suggesting that Bcl-2 negatively regulates Bax-mediated apoptosis [44,45]. In the present study, isoflurane anesthesia not only increased BAD expression and decreased Bcl-2 expression, but it also increased levels of the phosphorylated (active) p-JAK2 and p-STAT3 proteins. These results suggest that isoflurane-induced neural degeneration and apoptosis in elderly mice involve changes in Bcl-2 and BAD expression as well as activation of the JAK/STAT pathway. Dexmedetomidine pretreatment partially reversed the isoflurane-induced changes in BAD and Bcl-2 expression, while further increasing the levels of p-JAK2 and p-STAT3. Pretreatment with an α_2_ adrenoreceptor antagonist reversed the anti-apoptotic effects of dexmedetomidine, consistent with previous studies [7,8,10]. Similar results were obtained after pretreatment with a JAK2 or STAT3 inhibitor. Taken together, these results implicate Bcl-2 family members and the JAK2/STAT3 pathway in the anti-apoptotic effects of dexmedetomidine.

In addition to modulating synaptic function and plasticity, BDNF improves spatial learning and protects against memory impairment and neurodegeneration, in part by preventing neuronal death. Age-related decline in short-term and spatial working memory is associated with decreased BDNF expression in hippocampus; conversely, increasing BDNF expression improves both short and long-term memory and contributes to neuronal survival and differentiation [46,47]. Transcription of the BDNF gene is regulated by the CREB signaling pathway, which may help to explain why CREB has been linked to cell survival and differentiation, synaptic plasticity and long-term memory formation [48]. In the present study, isoflurane inhalation for 4 h reduced levels of BDNF and p-CREB in the hippocampus while also increasing apoptosis in that brain region and causing deficits in spatial learning and working memory, consistent with previous work [48]. A single dose of 50 μg/kg dexmedetomidine partially reversed all these effects of isoflurane, suggesting that dexmedetomidine works in part by increasing BDNF expression via the CREB signaling pathway. Similarly, dexmedetomidine pretreatment before intracerebral hemorrhage ameliorates the resulting impairment of short-term and spatial learning memory by suppressing apoptosis and enhancing BDNF expression [49].

Consistent with previous studies [10,30,38,47,50], our experiments suggest that prolonged exposure of senile mice to isoflurane caused spatial learning and memory deficits, neuronal apoptosis, and dramatic up-regulation of caspase-3, BAD in hippocampus, as well as down-regulation of Bcl-2, BDNF and p-CREB. Isoflurane exposure also promoted JAK2 and STAT3 activation in hippocampus accompanied with neurodegeneration in senile mice. Our results implicating the JAK2/STAT3 pathway in isoflurane-induced injury are consistent with the known importance of STAT3 for neuronal survival and cognitive formation[51,52]. JAK2-mediated tyrosine phosphorylation of STAT3 causes p-STAT3 to translocate to the nucleus, where it triggers transcription of target genes [53,54]. The JAK2/STAT3 pathway triggers expression of the anti-apoptotic protein Bcl-xL, which promotes survival of hippocampal neurons; this protective effect can be antagonized by AG490 or STAT3 decoy DNA, leading to mitochondrial membrane instability and mitochondrial oxidative stress [55,56]. In addition to activating BAD, isoflurane anesthesia may interfere with learning and memory function by activating the pro-apoptotic proteins Fas and FasL in hippocampus [57,58].

We examined the hippocampus only in the current study. However, it is unlikely that the observed changes are limited to the hippocampus. For example, a previous study shows that dexmedetomidine ameliorates frontal cerebral cortex impairment induced by brain hypoxia-ischemia injury in the neonatal rats [59]. Future studies are needed to examine the full range of apoptotic and signaling molecules affected by isoflurane and dexmedetomidine.

## Conclusions

Our behavioral and biochemical studies suggest that dexmedetomidine pretreatment could attenuate isoflurane-induced neurocognitive deficits in senile mice, possibly through the JAK2/STAT3 signaling pathway. If verified in patients, our results suggest that dexmedetomidine, already widely used clinically for sedation and analgesia, may also help prevent cognitive impairment after surgery in elderly patients.

## Supporting Information

S1 Table Escape latnecy during the training days: effects of isoflurane exposure and pretreatments: raw data. (XLSX)

S2 Table Swimming speed during the training days: effects of isoflurane exposure and pretreatments: raw data. (XLSX)

S3 Table Time spent in the third quadrant (S) and number of platform crossings during the probe testing: effects of isoflurane exposure and pretreatments: raw data. (XLSX)

S4 Table TUNEL-positive cells in the hippocampal CA1 region: effects of isoflurane exposure and pretreatments: raw data. (XLSX)

S5 Table Apoptosis proteins and BDNF in the hippocampus: effects of isoflurane exposure and pretreatments: raw data. (XLSX)

S6 Table JAK2/STAT3 pathway activation and p-CREB levels in hippocampus: effects of isoflurane exposure and pretreatments: raw data. (XLSX)

S7 Table Arterial blood gases and blood glucose: effects of isoflurane exposure and pretreatments: raw data. (XLSX)
